# Interception of Epoxide ring to quorum sensing system in *Enterococcus faecalis* and *Staphylococcus aureus*

**DOI:** 10.1186/s13568-023-01633-9

**Published:** 2023-11-09

**Authors:** Mohammed Y. Refai, Ahmed M. Elazzazy, Said E. Desouky, Mohammed Abu-Elghait, Eman A. Fayed, Sulaiman M. Alajel, Abdullah A. Alajlan, Mona O. Albureikan, Jiro Nakayama

**Affiliations:** 1 Department of Biological Science, College of Science, University of Jeddah, Jeddah, Saudi Arabia; 2 Laboratory of Microbial Technology, Division of Systems Bioengineering, Department of Bioscience and Biotechnology, Faculty of Agriculture, Graduate School, Kyushu University, 819-0395 Fukuoka, Japan; 3 Department of Botany and Microbiology, Faculty of Science, Al-Azhar University, 11884 Cairo, Egypt; 4 Department of Pharmaceutical Organic Chemistry, Faculty of Pharmacy (Girls), Al-Azhar University, 11754 Cairo, Egypt; 5 Reference Laboratory for Microbiology, Executive Department of Reference Laboratories, Research and Laboratories Sector, Saudi Food and Drug Authority (SFDA), Riyadh, Saudi Arabia; 6 Microbial Identification Division, Reference Laboratory for Microbiology, Executive Department of Reference Laboratories, Research and Laboratories Sector, Saudi Food and Drug Authority (SFDA), Riyadh, Saudi Arabia; 7 Department of Biological Sciences, Faculty of Science, King Abdulaziz University, 21589 Jeddah, Saudi Arabia

**Keywords:** *Enterococcus faecalis*, *Staphylococcus aureus*, Quorum sensing, *agr*, *fsr*, Epoxide ring ADMET and Docking Studies.

## Abstract

Quorum sensing inhibitor (QSI) has been attracting attention as anti-virulence agent which disarms pathogens of their virulence rather than killing them. QSI marking cyclic peptide-mediated QS in Gram-positive bacteria is an effective tool to overcome the crisis of antibiotic-dependent chemotherapy due to the emergence of drug resistance strain, e.g., methicillin resistant *Staphylococcus aureus* (MRSA) and Vancomycin resistant *Enterococci* (VRE). From a semi-large-scale screening thus far carried out, two Epoxide compounds, Ambuic acid and Synerazol, have been found to efficiently block *agr* and *fsr* QS systems, suggesting that the Epoxide group is involved in the mode of action of these QSIs. To address this notion, known natural Epoxide compounds, Cerulenin and Fosfomycin were examined for QSI activity for the *agr* and *fsr* systems in addition to *in silico* and SAR studies. As a result, most of investigated Epoxide containing antibiotics correlatively interfere with QSI activity for the *agr* and *fsr* systems under sublethal concentrations.

**Supplementary Information:**

The online version contains supplementary material available at 10.1186/s13568-023-01633-9.

## Introduction

Quorum sensing QS is a spirited system regulating the expression of a wide range of microbial genes (such as biofilm development, pathogenicity, and development of resistance and others) depending on cell density (Rutherford and Bassler, 2012; Saied et al., 2021; Waters and Bassler, 2005). The well-known core directing locus of the Gram-positive bacterial QS system is the accessory gene regulator (*Agr*) that has been considerably explored in *S. aureus* (El-gamal et al., 2017; Novick, 2003). while, in *E. faecalis, fsr* QS system has been discovered as a homolog of the *agr*-like QS system (Qin et al., 2000; Yousef et al., 2022). Even though Thiolactone- autoinducing peptides mediate the *agr* QS system, the *fsr* QS system is mediated by an activating peptide containing lactone called gelatinase biosynthesis-activating peptide (GBAP) (Nakayama et al., 2001). A Gram-positive pathogen *Staphylococcus aureus*, represents the most common reason for several diseases causing minor infections such as skin, and soft tissue infections, reaching life-threatening pneumonia, septicemia, endocarditis, and potentially fatal toxic shock (Said et al., 2023; Said et al., 2021; Shebl et al., 2021) (Desouky et al., 2014; Gordon and Lowy, 2008; Otto, 2012).

*S. aureus* infection is mediated via the *agr* locus by the release of several virulence agents and influencing biofilm formation. The *agr* locus involves two different units for transcription, RNAII and RNAIII, which are activated by the *agr* P2 and *agr* P3 promoters, respectively (Novick and Geisinger, 2008). The *agr* operon BDCA is encoded by RNAII, which is one of the QS structural components, while delta δ hemolysin is encoded by RNAIII which serves as a regulatory RNA implicated in a number of gene’s virulence (Le and Otto, 2015). Another type of opportunistic Gram-positive pathogen is *Enterococci* which lives as commensals in the gastrointestinal system. They commonly cause severe infections such as urinary tract infection, endocarditis, and bacteremia (Fiore et al., 2019; Lina et al., 1998). Gelatinase expression is positively controlled in the *fsr* locus by *fsr*A, *fsr*B, *fsr*C, and *fsr*D (Mylonakis et al., 2002). Nakayama (Nakayama et al., 2001) stated that the gelatinase synthesis which is regulated by the *fsr* QS has been associated with *Enterococci* pathogenicity. Methicillin-resistant *S. aureus* (MRSA) and vancomycin-resistant *Enterococci* (VRE) are among the most dangerous drug-resistant bacteria. Many antibiotics were shown to be ineffective against these bacteria, making therapeutic intervention much more difficult. The World Health Organization (WHO) expressed grave worry in 2017 about the progress of new antibiotics against diseases caused by multi-drug resistant (MDR) pathogens, including VRE and MRSA. The massive worldwide expansion of antimicrobial resistance, as well as a lack of effective and innovative medicines against these pathogens, may result in economic trouble and substantial morbidity in the future. (Molton et al., 2013; Prestinaci et al., 2015). Several MDR bacteria pathogenicity is regulated by the QS system. Consequently, QS interference has been proposed as an alternate strategy to these pathogens’ threats (Rasko and Sperandio, 2010; Rémy et al., 2018; Yousuf et al., 2018). Furthermore, one of the most recent attempts to develop anti-virulence medications that can lower virulence without being bactericidal is to target QS. (Defoirdt, 2018; LaSarre and Federle, 2013; Yousef et al., 2022). The need for wide spectrum antibiotics could be reduced when QS-inhibitors are utilized synergistically in combination with antibiotics or alone, and the chance of horizontal dissemination of drug-resistance genes is reduced (Baquero et al., 2011; Cegelski et al., 2008; Clatworthy et al., 2007). Several QS-inhibitors have been identified that exhibit promising therapeutic potential against Gram-positive bacteria pathogenicity *in vivo* and *in vitro* but they are still in experimental stages (Banhart et al., 2014; Daly et al., 2015; Gaber et al., 2020; Gaber et al., 2021; Nakayama et al., 2013). Previously, we reported a unique class of QS-inhibitors that drastically reduced *fsr* QS levels in *Enterococci*. (Nakayama et al., 2007; Nakayama et al., 2013). Lately, Desouky isolated cyclodepsipeptides as potent QS systems antagonists from actinomycetes microbial extracts, (Desouky et al., 2015). Accordingly, and as a part of our ongoing efforts to identify novel compounds with distinct mechanisms, here we scrutinize the inhibitory effects of a fungal secondary metabolite with the Epoxide group, Synerazol, which has been screened as a QSI compound. This discovery is intriguing because of its structure and activity association; Pseurotin A, a Synerazol analog, showed no QSI activity, whereas Synerazol inhibited the *fsr* and *agr* systems. The primary structural distinction between the two components is that Pseurotin A has a diol in place of Synerazol Epoxide at the same position. Also, a known QSI inhibitor, Ambuic acid, which inhibits both *agr* and *fsr* system through the inhibition of biosynthesis of auto-inducing peptide, has Epoxide. These structure-activity relationship data suggest that Epoxide group is involved in the mode of action of these QSI activities, which may target processing of autoinducing peptide precursor by the cysteine protease-like function of *Fsr*B/*Agr*B as known in the case of Ambuic acid and Cerulenin is a known antifungal antibiotic with a broad spectrum of inhibitory activities produced by *Cephalosporium caerulens* (Omura et al., 1967). Recently, many researchers around the world have been exploring alternatives to antibiotics in which new types created from natural or chemical sources are used to confront the disaster of multidrug resistance by targeting factors other than those targeted by the currently commercially available antibiotics (Abd Elkarim et al., 2021; Khattab et al., 2022; Okba et al., 2021; Soliman et al., 2022). To address this notion, in this study, other Epoxides including natural and unnatural compounds were examined for *agr* and *fsr* QSI activity. Furthermore, we employed molecular docking to explore the Epoxide group’s attraction for the *agr*A protein’s active site. This work adds to the repertory of Epoxide compounds that successfully inhibit QS system and provides more information toward the discovery of new antimicrobial drugs.

## Materials and methods

### Bacterial strains and culture media

Todd-Hewitt broth (THB) (Oxoid, UK) used to culture *E. faecalis* OG1RF (ATCC 47077), a gelatinase-positive strain, which is used to assess the QSI effects of the chemicals under examination. strain was cultivated at 37°C (Nakayama, Jiro et al., 2009). Finally, 7 µg/mL chloramphenicol added to the medium for cultivating *S. aureus* 8325- 4(pSB2035), for plasmid selection and grown in LB broth media (Oxoid, U K) and incubated at 37°C, with gentle agitation (Desouky et al., 2022) (Schmitt et al., 2012).

### **Assay for** ***E. faecalis fsr*** **QS system**

To measure fsr system inhibition, gelatinase activity in the culture supernatant of *E. faecalis* OG1RF grown with the tested drug was monitored. To address the inhibitory mode of action of samples, 0.5 McFarland (1.5 × 10^8^ CFU) of *E. faecalis* OU510 inoculated into THB medium containing the tested compounds (5µg) and cultivated at 37°C for 5 h with moderate agitation (Qin et al., 2000; Yousef et al., 2022). After measuring the growth turbidity at (OD600), the tube was centrifuged at 9,300 x g for 5 min, and 40 µl of supernatant were recovered. Azocoll reagent (Calbiochem, San Diego, CA) was used for the gelatinase assay, as previously reported. Briefly, the OD was measured at 540 after adding 40 µl of *E. faecalis* culture supernatant to 0.8 ml of Azocoll solution and mixing the mixture at 170 rpm for four hours. The evaluated compounds were applied to grown cells as a negative control sample, and the inhibitory impact was assessed by contrasting the inhibitory effects of the two groups of compounds. A compound that inhibits growth by at least 50% is considered significant and is evaluated for further study. A triplicate experiment was conducted.

### **Assay for** ***S. aureus agr*** **system**

Monitoring chemoluminescence of *S. aureus* 8325- 4(pSB2035) reporter strain, was used to evaluate *agr* inhibition. 0.5 McFarland standard overnight culture of *S. aureus* was then diluted 1: 50 in LB broth with different concentrations of the tested compounds, and then grown in a microtiter plate (96 wells) with 100 rpm shaking. For controls, the strains were grown without tested compounds. After 7 hours, the culture turbidity OD was measured at 620 nm using a reader (Immuno Mini NJ-2300; Nihon InterMed, Tokyo, Japan).

The bioluminescence was measured using a luminescence analyzer (LAS-4000mini; Fuji Photo Film, Japan). The induction level of luciferase was calculated by removing the fluorescence of the negative control. The total induction level was estimated by subtracting the brightness of the negative control from that of the positive one. A significant repressing impact was defined as one that was greater than 90%. The cells were extracted by centrifugation 9,300 x g for 2 minutes, washed twice in phosphate buffered saline (PBS), and then resuspended in 600µl of PBS.

### Docking protocol

The software of Molecular Operating Environment (MOE®) MOE 2014.0901 was used for molecular docking simulation investigation (El-Kalyoubi et al., 2022; Fayed et al., 2022; Fayed et al., 2021a; Fayed et al., 2021b; Ibrahim et al., 2021). Protein Data Bank was used to get the crystal structures of *S. aureus agr* A (PDB code: 3BS1). ChemDraw 2019 was used to create the 2D structures of the six chemicals Cerulenin, Fosfomycin, 1,2-epoxyethylbenzene, 1,2-epoxybutane, 2,3-epoxypropyl phenyl ether, Glycidyl methacrylate, and Ambuic acid. The protonated 3D was then constructed with the MOE 2014.0901 software using standard bond angles and lengths, followed by geometry optimization and energy minimization using the Conf Search module in MOE, and finally, the MOE files were saved to be available for the docking process. Using the Protonate 3D technique in MOE with the default settings, the structure of *agr* A was produced for docking (Leonard et al., 2012). In the docking protocol, the Triangle Matcher placement method and London dG scoring function were used.

### Statistical analysis

Triple duplicates were achieved for each experiment, and the results were the means of three different tests. To compare the distinctions between a sample and its corresponding control, the student’s t-test was performed. The differences were determined significant if the *p* values were less than 0.05.

## Results

### **Effect of natural Epoxide compounds on** ***E. faecalis***

**Fig. 1:**
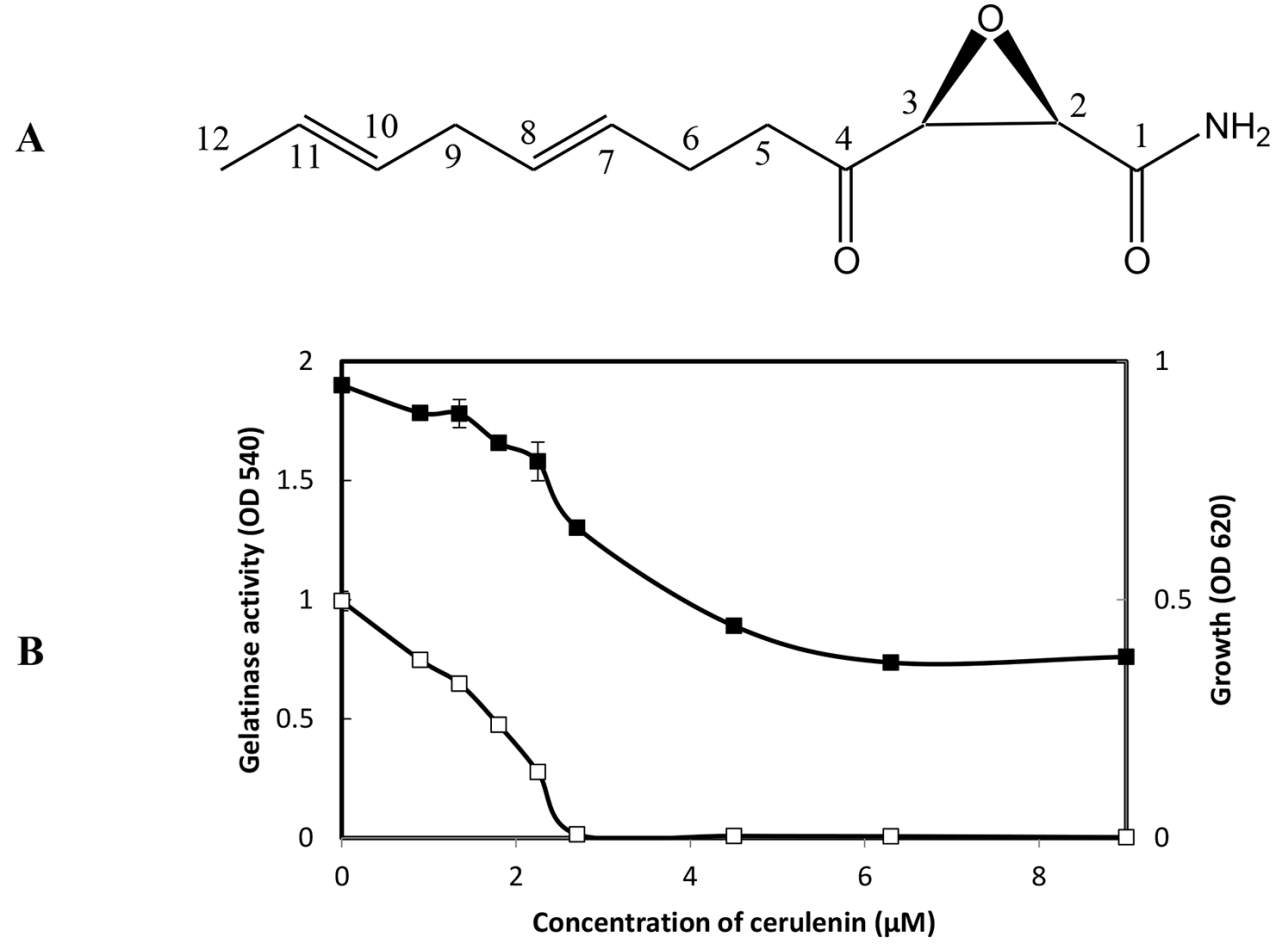
**A:** The chemical structure Cerulenin. **B:** Effect of cerulenin on *E. faecalis* OG1RF growth and gelatinase production. *E. faecalis* was grown in the presence or absence of cerulenin at the designated concentrations; the density of culture measured at the OD_620_ (closed square), and gelatinase activity was measured at OD_540_ (open square), the test was performed, and data represented as an average of triplicate experiments, ± standard deviation.

Since the QS *fsr* system consists of four genes responsible for the accumulation of gelatinase activating pheromones, detecting the presence or absence of the gelatinase enzyme was an indicator of the activity or inactivity of the quorum sensing system. Therefore, in this study, gelatinase was detected quantitatively using the Azocoll reagent, and the results were as follows:

Cerulenin structure is (2*R*) (3*S*)-2,3-epoxy-4-oxo-7,10- dodecadienoylamide (Fig. 1A) (Adhikari, R. P. and Novick, R. P., 2005; Goldberg, I. et al., 1973). In this study, it showed only slight growth inhibitory activity against *E. faecalis* at high concentrations. Like Synerazol, Cerulenin did not show direct inhibitory activity against GelE. On the other hand, at sublethal concentrations, Cerulenin substantially inhibited gelatinase synthesis mediated by the *fsr* QS system of MIC = 2.2 µM (Fig. 1B). However, IC_50_ dose (1.8 µM) of Cerulenin partially inhibited the gelatinase production in *E. faecalis* OU510 in the presence of physiological concentration (10 nM) of GBAP, suggesting that Cerulenin inhibits GBAP signal transduction more than GBAP biosynthesis.

**Fig. 2:**
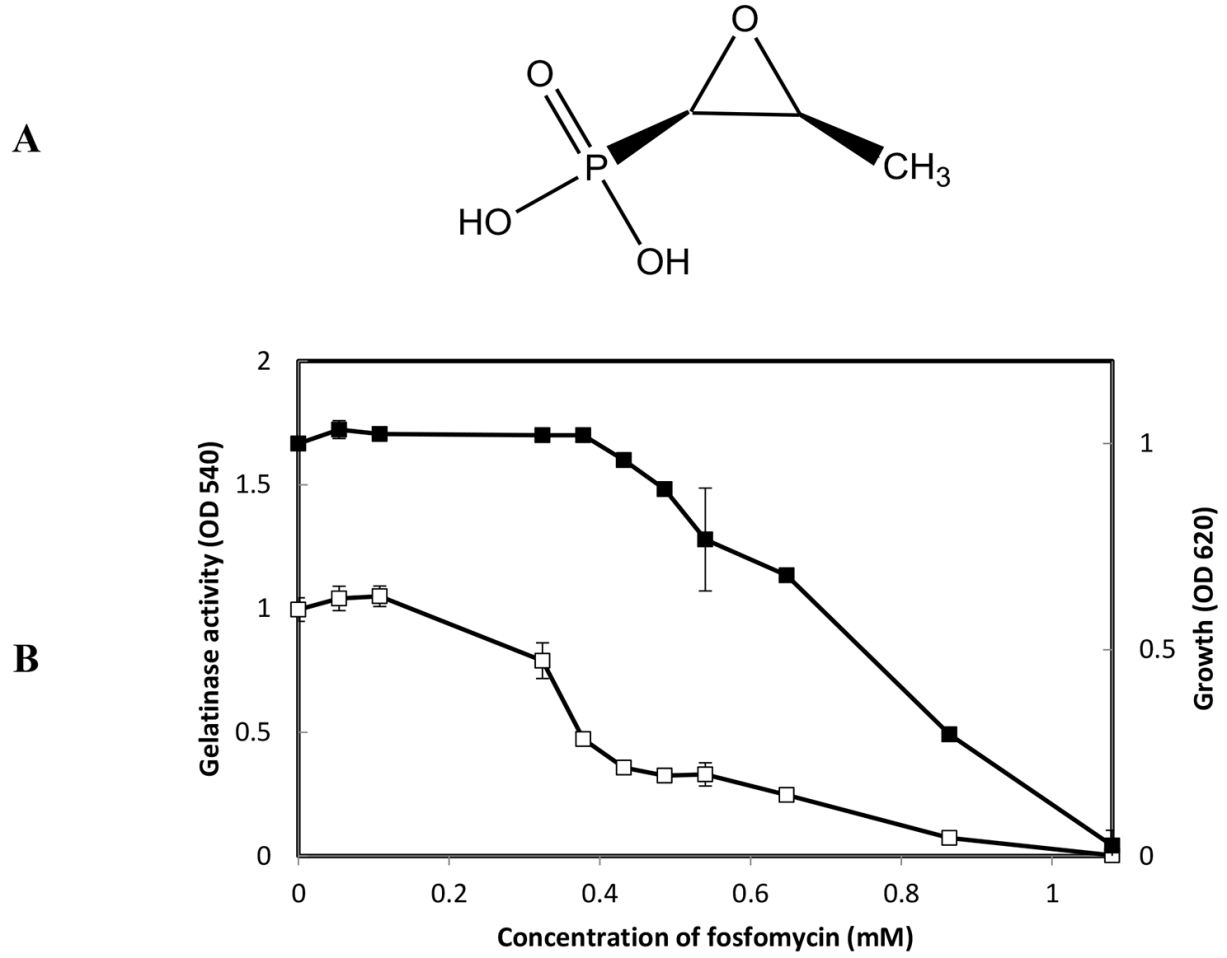
**A:** The chemical structure of fosfomycin. **B:** Effect of fosfomycin on *E. faecalis* OG1RF growth and gelatinase production. *E. faecalis* in the presence or absence of fosfomycin at the designated concentrations; the density of culture measured at the OD_620_ (closed square), while gelatinase activity at OD_540_ (open square), the test was performed and data represented as an average of triplicate experiments, ± standard deviation.

Fosfomycin (Kahan et al., 1974) is a known anti-Gram-positive antibiotics including MRSA (Grif, K. et al., 2001) and VRE (Superti et al., 2009) (Fig. 2A). In this study, it showed only slight growth inhibitory activity against *E. faecalis* at high concentrations. Like Synerazol, Fosfomycin did not show direct inhibitory activity against GelE. On the other hand, at sublethal concentrations, Fosfomycin substantially reduced gelatinase synthesis mediated by the *fsr* QS system where IC_50_ = 380 µM (Fig. 2B).

### **Effect of synthetic Epoxide compounds on** ***E. faecalis***

**Fig. 3:**
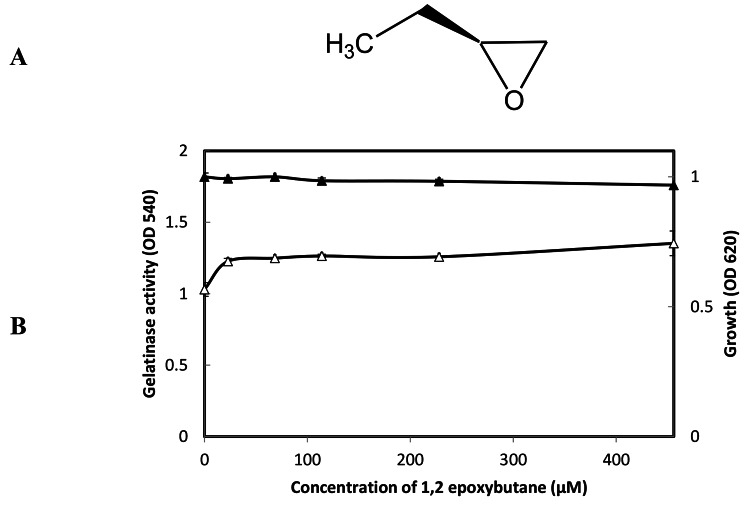
**A:** The chemical structure 1,2-epoxybutane. **B:** Effect of 1, 2-epoxybutane on *E. faecalis* OG1RF growth and gelatinase production. *E. faecalis* was grown in the presence or absence of 1, 2-epoxybutane at the designated concentrations; the density of culture measured at the OD_620_ (closed square), and gelatinase activity was measured at OD_540_ (open square), the test was performed, and data represented as an average of triplicate experiments, ± standard deviation.

1,2-epoxybutane, unnatural Epoxide compound (Fig. 3A), did not show effect on growth as well as gelatinase production in *E. faecalis* OG1RF (Fig. 3B).

**Fig. 4:**
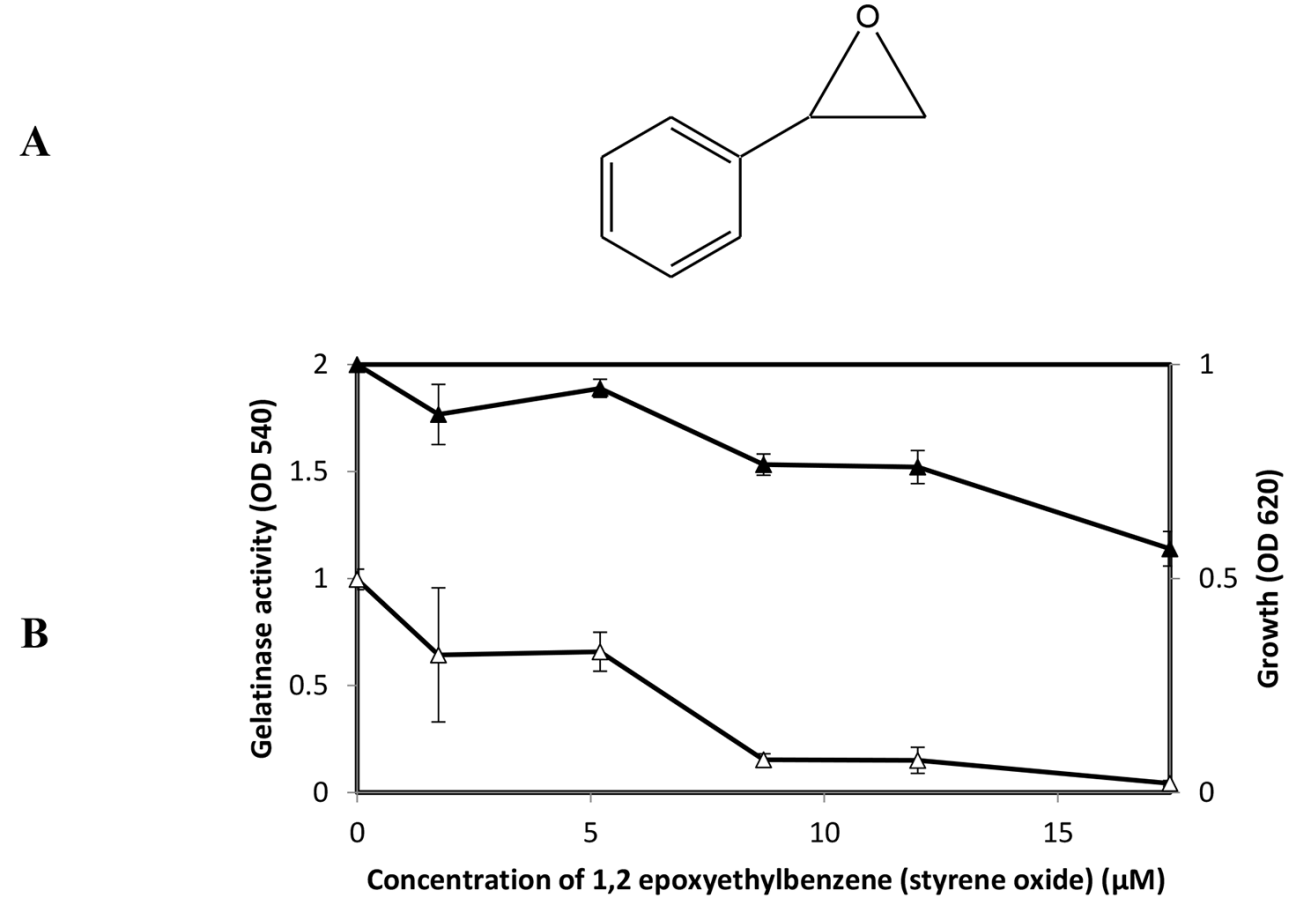
**A:** The chemical structure 1, 2-epoxyethylbenzene [49]. **B:** Effect of 1, 2-epoxyethylbenzene on *E. faecalis* OG1RF growth and gelatinase production. *E. faecalis* was grown for 5 h in the presence or absence of 1, 2-epoxyethylbenzene at the designated concentrations; the density of culture measured at the OD_620_ (closed square), and gelatinase activity was measured at OD_540_ (open square), the test was performed, and data represented as an average of triplicate experiments, ± standard deviation.

1,2-epoxyethylbenzene, styrene oxide, (Fig. 4A) showed only slight growth inhibitory activity against *E. faecalis* at those concentrations (Fig. 4B). As same as Synerazol, Cerulenin, 1,2-epoxyethylbenzene did not show direct inhibitory activity against GelE. On the other hand, 1,2-epoxyethylbenzene strongly inhibited the gelatinase production controlled by the *fsr* QS system at its sublethal concentrations of MIC = 13 µM. However, IC_50_ dose (6 µM) of 1, 2-Epoxyethylbenzene partially inhibited the gelatinase production in *E. faecalis* OU510 in the presence of physiological concentration (10 nM) of GBAP, suggesting that 1,2-Epoxyethylbenzene inhibits GBAP signal transduction more than GBAP biosynthesis.

**Fig. 5:**
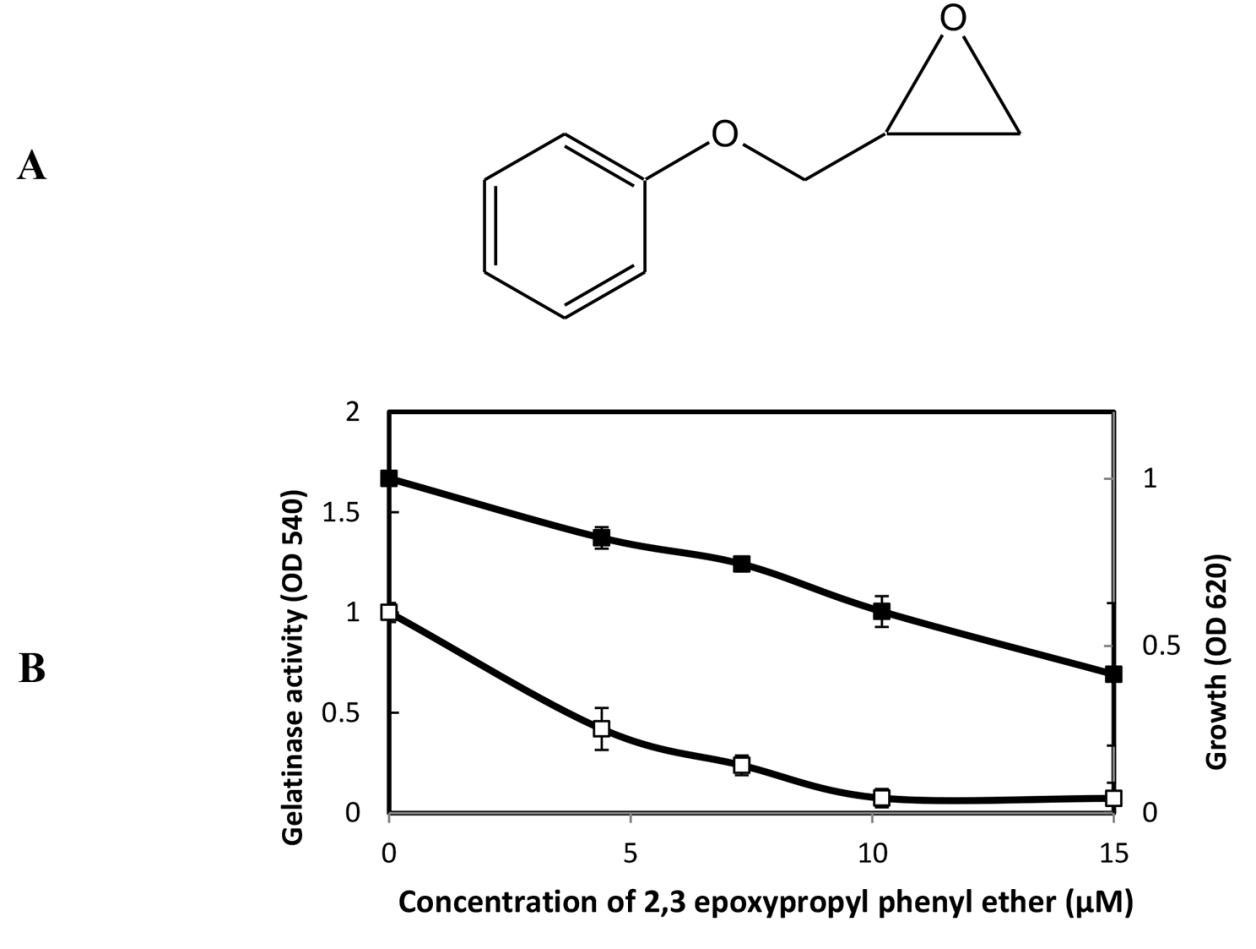
**A:** The chemical structure of 2,3-epoxypropyl phenyl ether [50]. **B:** Effect of 2, 3-epoxypropyl phenyl ether on *E. faecalis* OG1RF growth and gelatinase production. *E. faecalis* was grown for 5 h in the presence or absence of 2, 3-epoxypropyl phenyl ether at the designated concentrations; the density of culture measured at the OD_620_ (closed square), and gelatinase activity was measured at OD_540_ (open square), the test was performed, and data represented as an average of triplicate experiments, ± standard deviation.

In current study, 2,3-epoxypropyl phenyl ether (Fig. 5A) showed only slight growth inhibitory activity against *E. faecalis* at those concentrations (Fig. 5B). As same as Synerazol and Cerulenin, 2,3-epoxypropyl phenyl ether did not show direct inhibitory activity against GelE. On the other hand, 2,3-epoxypropyl phenyl ether strongly inhibited the gelatinase production controlled by the

*fsr* QS system at its sublethal concentrations of MIC = 10 µM. However, IC_50_ dose (6 µM) of 2,3-epoxypropyl phenyl ether partially inhibited the gelatinase production in *E. faecalis* OU510 in the presence of physiological concentration (10 nM) of GBAP, suggesting that 2,3-epoxypropyl phenyl ether inhibits GBAP signal transduction more than GBAP biosynthesis.

**Fig. 6:**
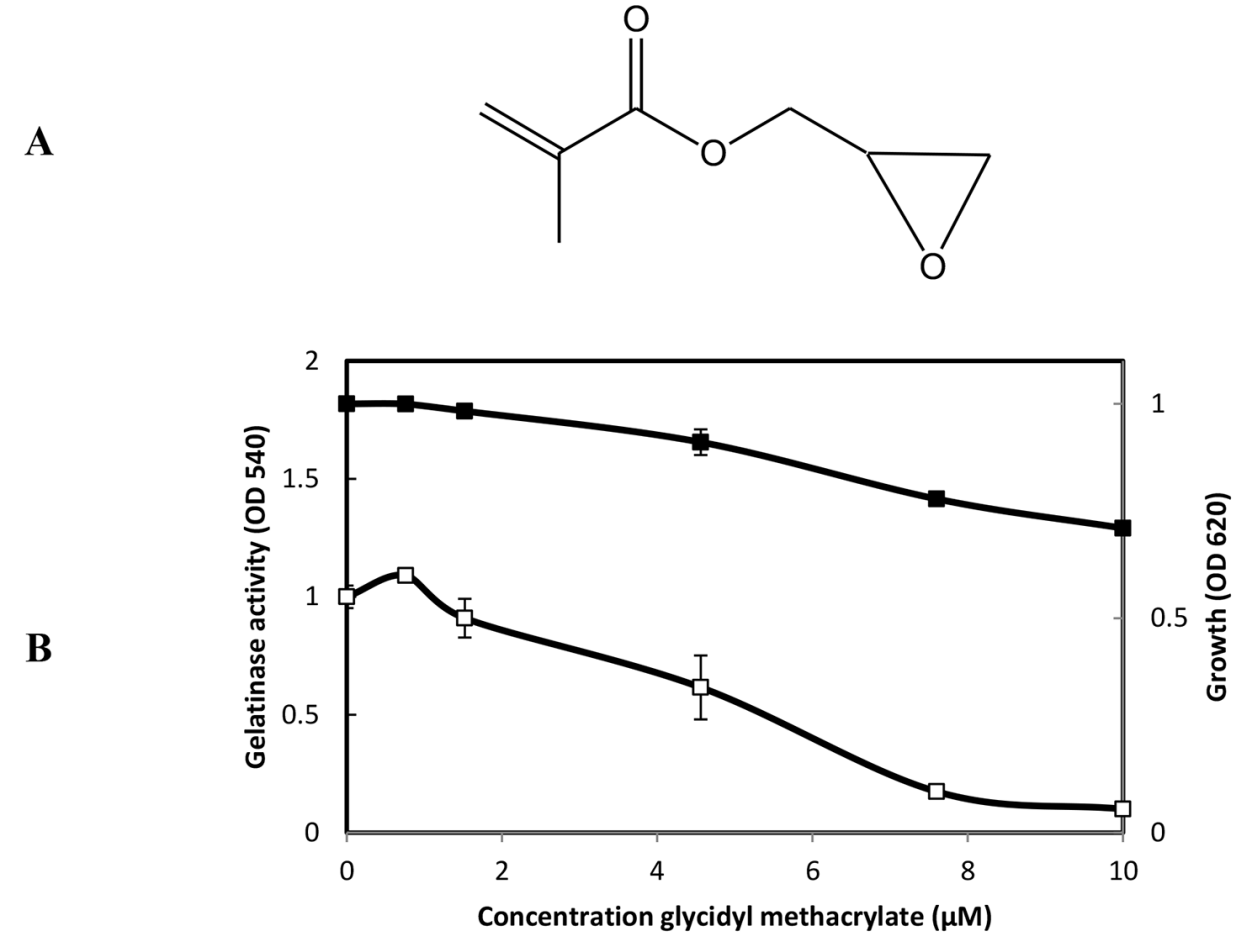
**A:** The chemical structure of glycidyl methacrylate [49]. **B:** Effect of glycidyl methacrylate on *E. faecalis* OG1RF growth and gelatinase production. *E. faecalis* was grown for 5 h in the presence or absence of glycidyl methacrylate at the designated concentrations; the density of culture measured at the OD_620_ (closed square), and gelatinase activity was measured at OD_540_ (open square), the test was performed, and data represented as an average of triplicate experiments, ± standard deviation.

**Fig. 7:**
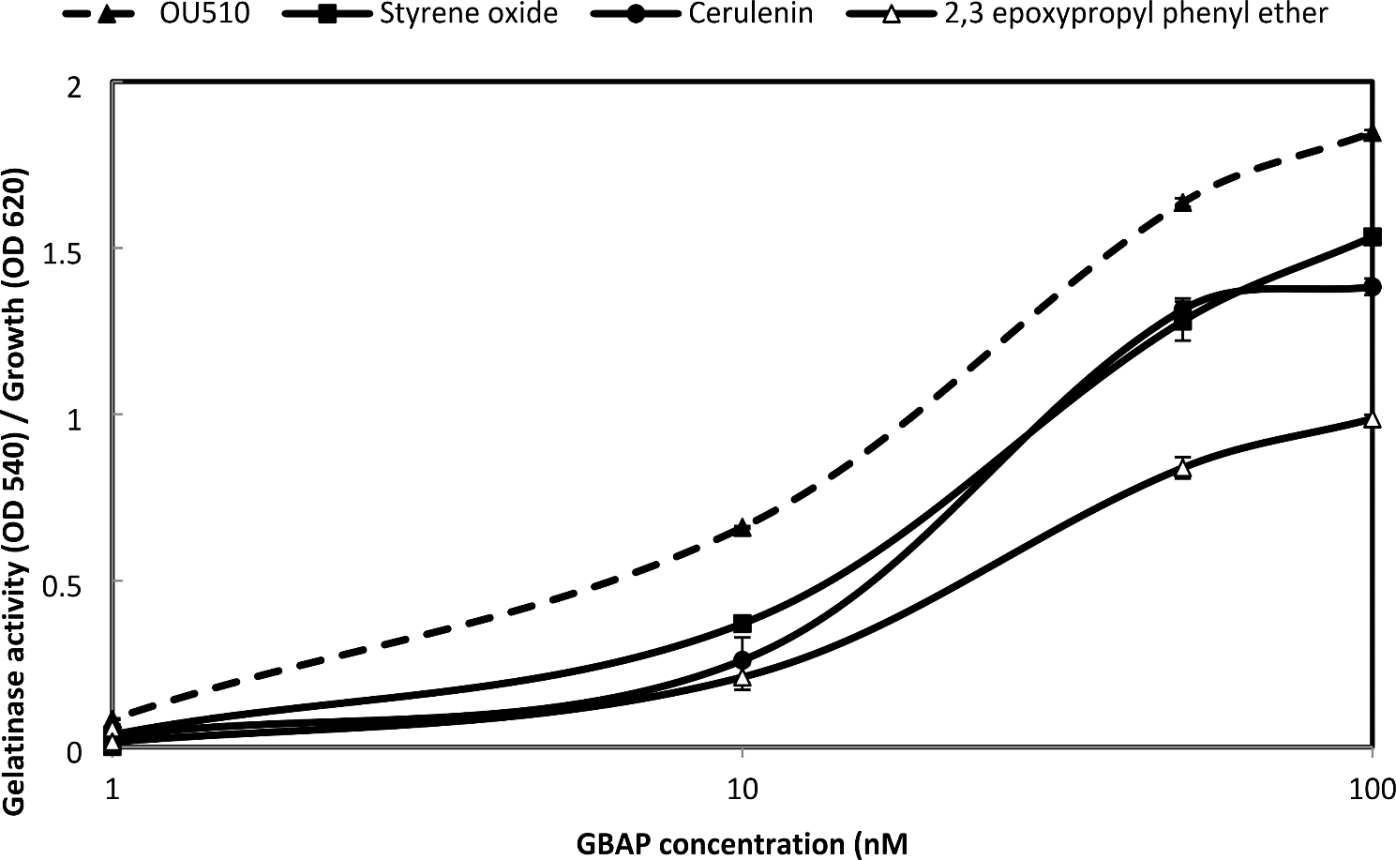
Effect of IC_50_ for QSIs on *E. faecalis* OU510 gelatinase production. This strain was induced by GBAP. *E. faecalis* OU510 was grown for 5 h in the presence of the designated concentrations of GBAP without QSIs (closed triangle with dashed line) or with 0.4 µM synerazol (open squares), 1.8 µM cerulenin (closed circle), 6 µM styrene oxide (closed square), and 4 µM 2, 3-epoxypropyl phenyl ether (open triangles). the test was performed, and data represented as an average of triplicate experiments, ± standard deviation.

Glycidyl methacrylate (Fig. 6A) showed only slight growth inhibitory activity against *E. faecalis* at those concentrations (Fig. 6B). As same as Synerazol, Cerulenin, and 2,3 epoxypropyl phenyl ether, glycidyl methacrylate did not show direct inhibitory activity against GelE. On the other hand, Glycidyl methacrylate strongly inhibited the gelatinase production controlled by the *fsr* QS system at its sublethal concentrations of MIC = 10 µM (Fig. 7).

### **Effect of Epoxide compounds on** ***agr*** **expression in** ***S. aureus***

**Fig. 8:**
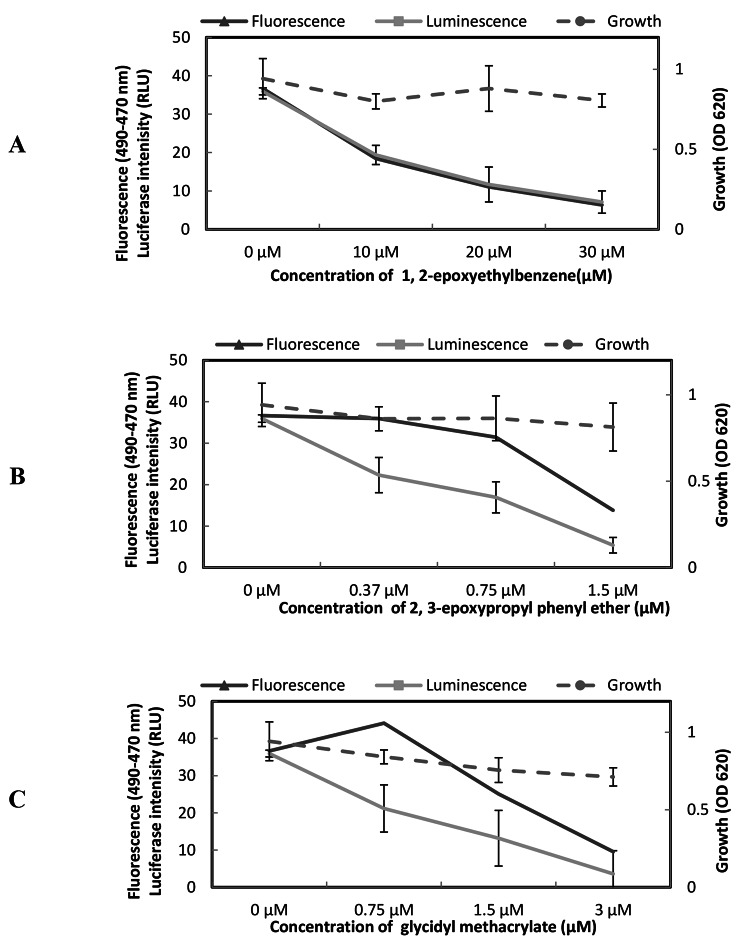
Effect of compounds on *S. aureus* 8325–4(pSB2035) *agr* expression in. The dual reporter strain grew in the presence or absence of various concentrations of compounds. After 7 h, the OD_620_, fluorescence at 490 nm and 470 nm and chemiluminescence of the culture were measured. **A:** 1, 2-epoxyethylbenzene, **B:** 2, 3-epoxypropyl phenyl ether, **C:** glycidyl methacrylate.

1,2-epoxyethylbenzene (Fig. 8A), 2,3-epoxypropyl phenyl ether (Fig. 8B), and glycidyl methacrylate **(**Fig. 8C) inhibited the expressions of both GFP and luciferase in dose-dependent manner with IC_50_ for GFP expression approximately equal to 10, 1, and 1.5 µM, respectively and IC_50_ for luciferase expression approximately equal to 15, 0.75, and 1.5 µM, respectively.

### In silico Studies

#### Assessment of physicochemical and ADME properties

SwissADME was used to assess the physicochemical and ADME properties of Cerulenin and Fosfomycin (Adhikari, Rajan P and Novick, Richard P, 2005; ANDO et al., 1991; Forsgren, Arne and Walder, Mats, 1983; Goldberg, Israel et al., 1973; Program, 1988). According to physicochemical metrics, all the substances tested met Lipinski’s standards for oral pharmaceuticals (Table S1). Furthermore, according to Veber’s standards, all the compounds meet the drug-likeness criterion. All the hits have rotatable bonds ranging from 1 to 7, indicating that their molecular target is adaptable. Table 1 shows that the total topological polar surface area (TPSA) is less than 115 A^0 2^. Additionally, absorption (percent ABS) was determined using the equation% ABS = 109 - (0.345 x TPSA) (Grif, Katharina et al., 2001), and the calculated percent ABS of all hits ranged from 81.44 to 104.68%, indicating that these derivatives may have the needed cell membrane permeability and bioavailability.

**Table 1:** Physicochemical properties based on TPSA, and % ABS.

Compounds	TPSA	% ABS
Cerulenin	72.69	83.92
Fosfomycin	79.87	81.44
Glycidyl methacrylate	38.83	95.60

all tested hits have significant-high gastrointestinal absorption in relation to the pharmacokinetic and medicinal chemistry parameters. Cerulenin and Fosfomycin show no permeability of the blood-brain barrier and there are no CNS side effects (Table S2).

Another trait that distinguishes P-glycoprotein (P-gp) is its non-substrate candidature. P-gp is an efflux transporter that moves medicines, other constituents, and their substrates out of cells. As a result, the substances were investigated via the SwissADME website. As indicated in Table 1, all chemicals are P-gp protein substrates, indicating that these hits have a very low likelihood of effluxing out of the cell, resulting in a maximal effect.

The most essential factor determining absorption is bioavailability, which is a measurement of the amount of drug in the bloodstream. Surprisingly, all the hits had high bioavailability levels between 0.55 and 0.56. All of the compounds had zero warnings, according to SwissADME’s Pan Assay Interference Compounds (PAINS) (Adhikari, Rajan P and Novick, Richard P, 2005). Though PAINS are important criteria to deliberate when creating drugs to reduce false-positive results, overestimating and applying these filters blindly can lead to the rejection of potential achievements based on phantom PAINS (Igarashi et al., 2004). All the compounds have synthetic accessibility ratings ranging from 1.00 to 3.93, indicating that they can be easily synthesized on a wide scale.

### Toxicity prediction

Safety is always the most significant factor throughout the development of pharmaceutical agents, which includes a wide spectrum of toxicities and adverse pharmacological properties that should be examined throughout the clinical trial and preclinical stages.

a wide range of toxicities and adverse pharmacological effects that should be investigated during the preclinical and clinical trial phases. So, the toxicity of all compounds Cerulenin and Fosfomycin, was investigated using the web tools pkCSM (http://biosig.unimelb.edu.au/pkcsm/prediction) and ProTox-II (https://tox-new.charite.de/protox II) (Han et al., 2019; Kamoutsis et al., 2021). Cerulenin and Fosfomycin showed AMES toxicity, with maximum tolerated human doses equal 0.473 and 1.490 log mg/kg/day, respectively as well as values for oral rat acute toxicity (LOAEL) were 2.015 to 2.925 mol/kg, respectively and oral rat chronic toxicity values equal 2.850 and 2.517 log mg/kg_ bw/day) respectively according to the pkCSM web tools.

Hepatotoxicity is a mutual reason for pharmaceutical withdrawal from clinical trials and manufacturing. In vitro experiments can be performed on immortalized cell lines such as HepG-2, primary human hepatocyte cultures, and liver slices.

Due to ProTox-II detection, they were non-immunotoxic and inert against phosphoprotein p53 and cytotoxicity, according to the toxicity profile, as shown in the preceding table (Table S3). Cerulenin is likely to be mutagenic. Cerulenin was classified as class four (GHS), but Fosfomycin was classified as group five (Table S3).

### Molecular docking studies

**Fig. 9:**
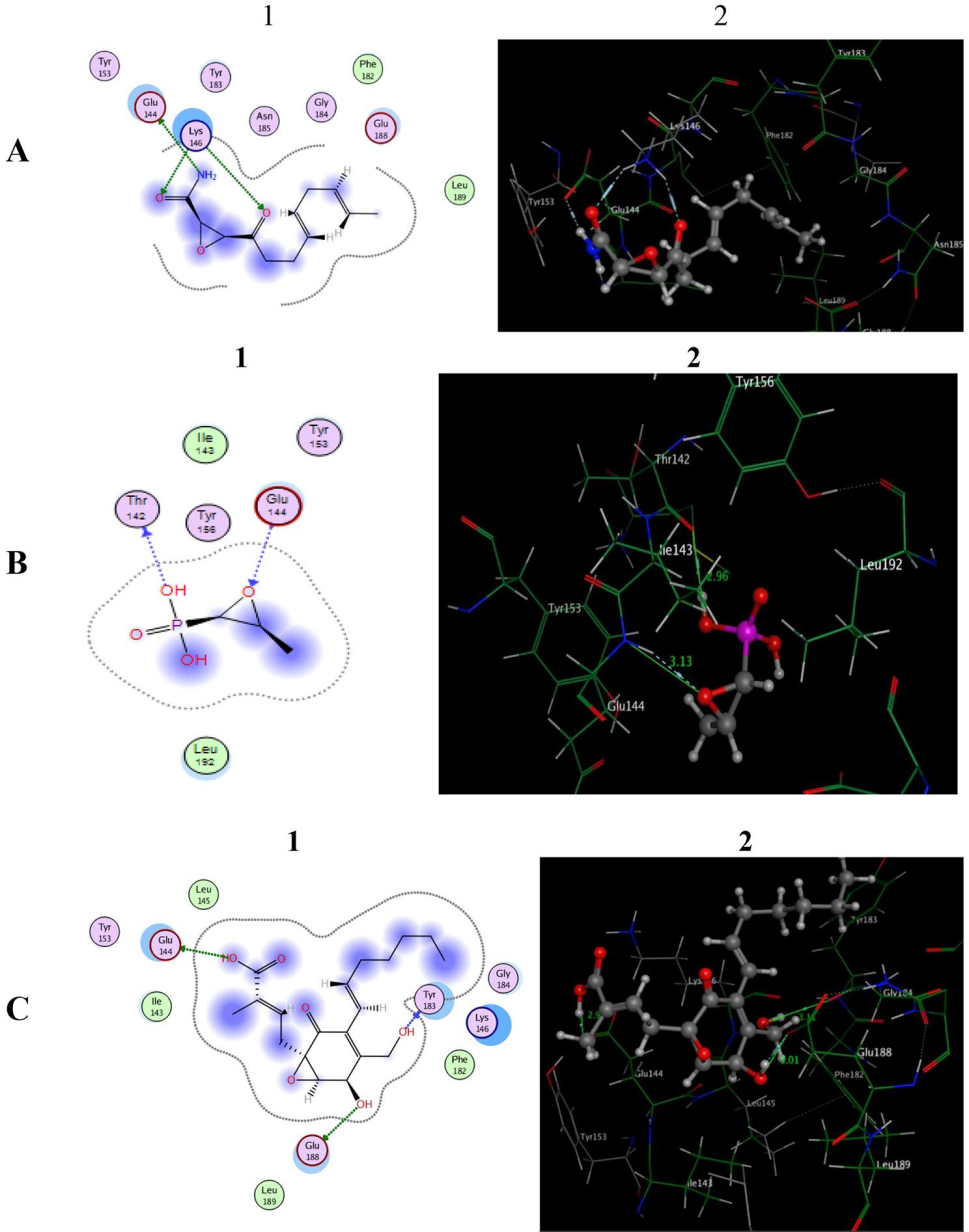
The two dimensional (1) and three-dimensional (2) interacting mode of **A:** Cerulenin **B:** Fosfomycin, **C:** Ambuic acid in the active region of AgrA (PDB code: 3BS1).

As depicted in Fig. 9A, Cerulenin formed a hydrogen bond donor between the nitrogen of the amidic group and the Side chain of **Glu144** (distance: 3.16 Å). Moreover, the oxygen of the two carbonyl groups shared fixation with the sidechain of **Lys146** via two H-bond acceptors (distance: 2.93, 3.12 Å, respectively).

Figure 9B shows how the epoxy group of the Fosfomycin scaffold kept its increased potency by forming hydrogen bonds between the oxygen atom and the backbone of the **Glu144** residue (distance: 3.13 Å). Furthermore, the phosphate hydroxy group established an H-bond donor with the backbone of **Thr142** (distance: 2.96 Å).

Finally, the Ambuic acid was docked into the active site of *agr*A which represented three hydrogen bonds with three amino acids. Two hydrogen bond donors, the first is the H-bond of the oxygen atom of the carboxylic group with the sidechain of **Glu144** while the second is for the phenolic oxygen with the sidechain of **Glu188** (distance: 2.95, 3.01 Å), respectively. Moreover, the alcoholic oxygen of Ambuic acid showed a hydrogen bond acceptor with the backbone of **Tyr183** (distance: 3.11 Å) (Fig. 9C).

#### SAR study

From the previous work (Capuzzi et al., 2017), Synerazol has been reported to function as a universal regulator of the *fsr*/*agr* QS systems. The stronger Synerazol’s inhibitory effect might be related to the presence of an Epoxide fragment, which is significant in the binding site of the essential amino acid in *Agr*A. Here we tested a series of natural and unnatural compounds that contain Epoxide rings. The results indicated Epoxide-containing compounds had moderate to high activity as QSI targeting *fsr* and *agr* system, according to the prior findings. Based on the structural composition of the substances, all contain Epoxide ring which is essential in the binding with the amino acids. Fosfomycin and Ambuic acid show binding of the oxygen atom of Epoxide with **Glu144** with a high energy score except Cerulenin, which contains additional functional groups which make 3 H-bonds with two amino acids which are, **Glu144** and **Lys146**. Here the amino acid **Glu144** formed a hydrogen bond donor between the nitrogen of the amidic group and its sidechain and not the oxygen atom of Epoxide with a high energy score (-8.697 kcal/mol) and Root mean square deviation (RMSD) equal 1.731 and it is the most suitable conformer of the structure during the docking process. The higher activity of this compound may be due to the presence of other functional groups such as two C = O, and NH_2_, which can form additional bonds with other amino acids of the enzyme.

In addition, Ambuic acid showed a high binding energy score (-10.39 kcal/mol) and RMSD equal 1.609 as it contains three hydroxy groups that can form three hydrogen bonds with the essential amino acids. It formed two hydrogen bonds donor for carboxylic and phenolic oxygen with the sidechain of Glu144 and Glu188, respectively, while its alcoholic oxygen formed a hydrogen bond acceptor with the backbone of Tyr183. So, we need to study a large group of Epoxide-containing compounds of different sizes and additional functional groups to establish the effect of the Epoxide ring as *agr*/*fsr* quorum sensing inhibitors for *Staphylococcus aureus* and *E. faecalis.*

## Discussion

From relatively small-scale screening, Nakayama found Siamycin and Ambuic acid as QSIs targeting *Enterococcal fsr* quorum sensing. Interestingly, these two compounds showed different modes of action. Siamycin inhibits the *fsr* QS system of *E. faecalis* via the block of *Fsr*C-*Fsr*A two component signal transduction. On the other hand, Ambuic acid inhibits the biosynthesis of cyclic peptide autoinducer of *Listeria innocua* and *S. aureus* (Nakayama, J. et al., 2009), which resulted in wide-spectral QSI activity. The biosynthetic mechanism of cyclic peptide autoinducers has been investigated for both *E. faecalis* and *S. aureus.* In both cases, it is supposed that *Fsr*B and *Agr*B are involved in the process and maybe in cyclization of precursor peptides in the cysteine protease-like manner because catalytic residue-like amino acids, cysteine, and histidine are conserved and essential for the biosynthesis of mature autoinducers. Even though Ambuic acid was suspected to target the cysteine protease-like function of *Fsr*B/*Agr*B, the precise inhibitory mechanism of Ambuic acid was unexplored on the molecular structure level.

Cerulenin, an antifungal antibiotic with a broad spectrum of inhibitory activities, acts as an inhibitor of fatty acid synthesis and related processes (Adhikari, R. P. and Novick, R. P., 2005; Goldberg, I. et al., 1973). In this study, Cerulenin inhibited gelatinase production induced by the *fsr* QS system in *E. faecalis.* The dose response curve of Cerulenin showed an interesting profile; it completely inhibited gelatinase production at the concentrations higher than 3 µM while the cell growth was also suppressed at the concentrations higher than 3 µM but still about a half of the maximum growth was seen at 10 µM. Furthermore, 30% was done at 1 mM. This bacteriostatic activity, which fails to abolish cell growth, is favorable in terms of avoidance of selective pressure to promote the emergence of drug resistant strain. On the other hand, Cerulenin enhanced *agr* expression in *S. aureus* in a dose dependent manner from 0 to 4 µM. However, in a previous study by Adhikari, R. P. and Novick, R. P. (2005), showed that Cerulenin strongly inhibited *agr* system at 5 and 10 µg per ml corresponding to approximately 20 to 40 µM. However, in our study, dose higher than 4 µM strongly inhibited the bacterial cell growth, and we could not discriminate QSI activity from growth inhibitory activity. Cerulenin is not like the case of Synerazol or Ambuic acid and may modulate directly or indirectly the functions of some different molecules other than those involved in *agr* QS system. Cerulenin is known to inhibit fatty acid synthesis by covalently binding to the cysteine residue of beta-keto-acyl-ACP synthase of not only eukaryote but also bacteria. It is also known that Cerulenin blocks ACP synthase activity resulting in the lack in the transfer of acyl chain to *N*-acyl homoserine lactone auto-inducer in Gram negative bacteria (Val and Cronan, 1998).

In this study, Cerulenin inhibited gelatinase production induced by fsr QS system in E. faecalis. The dose-response curve of Cerulenin showed an interesting profile; it completely inhibited gelatinase production at concentrations higher than 3 µM while the cell growth was also suppressed at concentrations higher than 3 µM but still about half of the maximum growth was seen at 10 µM. Furthermore, 30% was done at 1 mM. This bacteriostatic activity, which fails to abolish cell growth, is favorable in terms of avoidance of selective pressure to promote the emergence of drug-resistant strains. On the other hand, Cerulenin enhanced agr expression in S. aureus in a dose-dependent manner from 0 to 4 µM. However, a previous study by Adhikari, R. P. and Novick, R. P. (2005), showed that Cerulenin strongly inhibited the agr system at 5 and 10 µg per ml corresponding to approximately 20 to 40 µM. However, in our study, a dose higher than 4 µM strongly inhibited bacterial cell growth, and we could not discriminate QSI activity from growth inhibitory activity. Cerulenin is not like the case of Synerazol or Ambuic acid and may modulate directly or indirectly the functions of some different molecules other than those involved in the agr QS system. Cerulenin is known to inhibit fatty acid synthesis by covalently binding to the cysteine residue of beta-keto-acyl-ACP synthase of not only eukaryotes but also bacteria. It is also known that Cerulenin blocks ACP synthase activity resulting in the lack of the transfer of the acyl chain to N-acyl homoserine lactone auto-inducer in Gram-negative bacteria (Val and Cronan, 1998).

This mode of action is highly interesting since the biosynthetic enzyme of GBAP, *Fsr*B, has a catalytic cysteine residue which might be the target of Cerulenin. However, not only the cysteine residue of *Fsr*B but also that of many other enzymes including ACP synthase can be the target of Cerulenin. This complex mode may explain the partial growth inhibitory effect of Cerulenin but also the discrepancy in its effect on between *Enterococcus fsr* and *Staphylococcus agr.*

Fosfomycin is an antibiotics active against gram-positive bacteria including MRSA (Grif, K. et al., 2001) and VRE (Superti et al., 2009) by inhibiting cell wall synthesis as a mimic of phosphoenolpyruvate which is transferred to UDP-*N*-acetylglucosamine to generate UDP-*N*-acetylglucosamine pyruvate ether (Forsgren, A. and Walder, M., 1983). Similar to Cerulenin, Fosfomycin is also known to react with cysteine catalytic residue of UDP-*N*-acetylglucosamine-3-enolpyruvyltransferase.

In this study, more than 0.4 mM of Fosfomycin sharply affects growth pattern in *E. faecalis*, but under sub-lethal dose we found IC_50_ for blocking gelatinase production is 0.37 mM without influencing growth. It could be suspected that this blocking activity against *fsr* system is due to the covalently binding of Fosfomycin to the catalytic cysteine residue of *Fsr*B.

Regards the QSI effect of Synerazol which contains epoxy group as reported in our previous work (Igarashi et al., 2004). Here we decided to study compounds with the epoxy group to determine their QSI activities. So, the molecular docking simulation was used to investigate the binding modalities of the potent targets Cerulenin and Fosfomycin inside the active site of *S. aureus Agr*A (PDB code: 3BS1), this was performed using MOE program. Cerulenin, Fosfomycin and Ambuic acid were docked into the ATP-active region of *Agr*A, with energy scores of -8.69, -7.32, and − 10.39 kcal/mol, and RMSD of 1.731, 0.877, and 1.609 respectively. In conclusion, as a result of Epoxide-containing antibiotics investigation, Cerulenin and Fosfomycin interfered with QS activity for both the *agr* and *fsr* systems under the sublethal concentrations which makes these compounds a promising solution to the antibiotic-resistant problem.

### Electronic Supplementary Material

Below is the link to the electronic supplementary material

Supplementary Material 1

## Data Availability

All authors declare that the data supporting the findings of this study are available within the article.
