# Supplementary material for: Interception of Epoxide ring to quorum sensing system in Enterococcus faecalis and Staphylococcus aureus
Source: AMB Express. 2023 Nov 9;13:126. doi: 10.1186/s13568-023-01633-9 (PMC10636001; doi:10.1186/s13568-023-01633-9)
Supplement: Supplementary file 1 — Supplementary Material 1 [file 13568_2023_1633_MOESM1_ESM.docx]

**Table S1:** properties of physicochemical based on Lipinski´s rule of five, and number of rotatable bonds

| **Compounds** | **HBD**  **˂ 5** | **HBA**  **˂ 10** | **MlogP**  **˂ 4.15** | **MW**  **˂ 500** | **Lipinski´s Violations** | **Veber´s violations** | **No. of**  **rot.bonds** |
| --- | --- | --- | --- | --- | --- | --- | --- |
| **Cerulenin** | 1 | 3 | 0.33 | 223.27 | 0 | 0 | 7 |
| **Fosfomycin** | 2 | 4 | -1.68 | 138.06 | 0 | 0 | 1 |

**Table S2:** Pharmacokinetic properties and medicinal chemistry parameters

| **Compounds** | **GI**  **Absorption** | **BBB**  **Permeation** | **Pgp substrate** | **Bioavailability**  **Score** | **PAINS**  **Alerts** | **Synthetic**  **Accessibility** |
| --- | --- | --- | --- | --- | --- | --- |
| **Cerulenin** | High | No | No | 0.55 | 0 | 3.42 |
| **Fosfomycin** | High | No | No | 0.56 | 0 | 3.93 |

**Table S3:** Toxicity of the tested compounds

| **Test** | **Tested compounds** | |
| --- | --- | --- |
| **pkCSM prediction** | | |
| **Compounds** | **Cerulenin** | **Fosfomycin** |
| **AMES toxicity** | Yes | Yes |
| **Max. tolerated dose (human) (log mg/kg/day)** | 0.473 | 1.490 |
| **hERG I inhibitor** | No | No |
| **hERG II inhibitor** | No | No |
| **Oral Rat Acute Toxicity (LD_50_) (mol/kg)** | 2.015 | 2.925 |
| **Oral Rat Chronic Toxicity (LOAEL) (log mg/kg_ bw/day)** | 2.850 | 2.517 |
| **Hepatotoxicity** | No | Yes |
| **Skin Sensitization (log ug/L)** | No | No |
| **T. Pyriformis toxicity** | 0.571 | -0.202 |
| **Minnow toxicity (log mM)** | 1.466 | 2.098 |
| **ProTox-II prediction** | | |
| **LD_50_ mg/kg** | 547 | 3500 |
| **Toxicity Class** | IV | V |
| **Immunotoxicity** | Inactive 0.97 | Inactive 0.99 |
| **Mutagenicity** | Active 0.57 | Inactive 0.50 |
| **Cytotoxicity** | Inactive 0.67 | Inactive 0.71 |
| **Phosphoprotein**  **(Tumor Suppressor) p53** | Inactive 0.82 | Inactive 0.95 |
